# The Role of Phosphate-Solubilizing Microbial Interactions in Phosphorus Activation and Utilization in Plant–Soil Systems: A Review

**DOI:** 10.3390/plants13192686

**Published:** 2024-09-25

**Authors:** Ying Zhu, Yijing Xing, Yue Li, Jingyi Jia, Yeqing Ying, Wenhui Shi

**Affiliations:** 1 State Key Laboratory of Subtropical Silviculture, Zhejiang A&F University, Hangzhou 311300, Chinajiajingyi@stu.zafu.edu.cn (J.J.); 2 Key Laboratory of Bamboo Science and Technology, Zhejiang A&F University, Hangzhou 311300, China

**Keywords:** phosphorus limitation, phosphate-solubilizing microorganisms, microbial interactions, microbial function, synthetic communities (SynCom)

## Abstract

To address the issue of phosphorus limitation in agricultural and forestry production and to identify green and economical alternatives to chemical phosphorus fertilizers, this paper reviews the utilization of phosphorus in plant–soil systems and explores the considerable potential for exploiting endogenous phosphorus resources. The application of phosphate-solubilizing microorganisms (PSMs) is emphasized for their role in phosphorus activation and plant growth promotion. A focus is placed on microbial interactions as an entry point to regulate the functional rhizosphere microbiome, introducing the concept of synthetic communities. This approach aims to deepen the understanding of PSM interactions across plant root, soil, and microbial interfaces, providing a theoretical foundation for the development and application of biological regulation technologies to enhance phosphorus utilization efficiency.

## 1. Introduction

**Figure 1:**
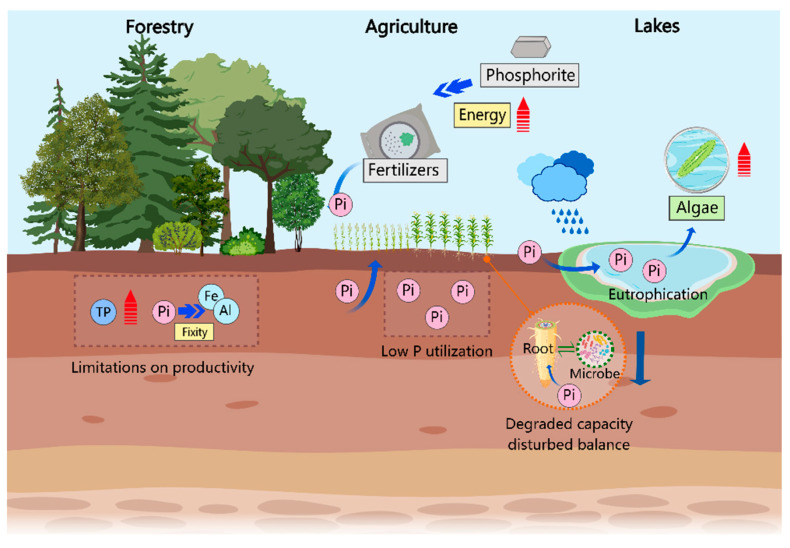
Phosphorus limitation in production and common solutions.

Phosphorus (P) in soil is primarily absorbed and utilized by plants in the form of orthophosphate (Pi), participating in nearly all major metabolic processes and significantly influencing plant productivity levels [1]. Therefore, an adequate supply of Pi in the soil is not only a prerequisite for the maintenance of plant growth and development but also a crucial factor in ensuring agricultural production [2] (Figure 1). However, Pi in the soil is easily fixed by other mineral elements (such as calcium, aluminum, and iron), resulting in soil solution concentrations of Pi that are generally lower than plant requirements, thereby affecting crop yield and quality [3]. To overcome phosphorus deficiency, chemical phosphorus fertilizers are applied, but their utilization rate within the same season is extremely low, less than 30% [4,5], leading to their frequent overuse. Long-term and high-frequency excessive application of phosphorus fertilizers results in residual phosphorus nutrients entering nearby water bodies through surface runoff or leaching, causing eutrophication, promoting algal blooms, and subsequently disrupting aquatic ecosystems, reducing human food sources, and posing risks to food security [6,7,8]. Excessive use of phosphorus fertilizers can also inhibit root growth [9,10], disrupt plant–microbial interactions [11,12], reduce the effectiveness of trace soil nutrients [13], and deteriorate the rhizosphere microecological environment, thereby limiting nutrient efficiency and affecting plant growth and development. Additionally, phosphate rock resources are non-renewable, and global reserves are depleting rapidly, expected to be exhausted within this century, insufficient to meet the increasing demand for phosphorus fertilizers sustainably and at high quality, with rising mining costs and high energy consumption for fertilizer synthesis, which will likely lead to increased phosphorus fertilizer prices, limiting food production [8,14].

These issues highlight the limited prospects for the development and use of phosphorus fertilizers and the potential ecological, environmental, and food security challenges that cannot be ignored. Therefore, to reduce the excessive reliance on chemical phosphorus fertilizers, it is necessary to scientifically control the levels of available phosphorus in agricultural soil systems [15] and to seek environmentally friendly and economically viable innovative approaches to enhance the transformation and utilization efficiency of phosphorus in subterranean systems.

To meet plant growth and production needs and to effectively harness the potential of rhizobiology, the functionality of the rhizosphere-promoting microbial community can be adjusted [16], enhancing the levels of available phosphorus in the rhizosphere and increasing root phosphorus absorption and utilization, thereby achieving scientific management of soil phosphorus. Among the rhizosphere-promoting microbes, a group known as phosphate-solubilizing microorganisms plays a significant role in the activation and utilization of soil phosphorus [17]. Current research on phosphate-solubilizing microorganisms primarily focuses on the solubilizing functions of individual strains [18,19] and their molecular interactions with plants [20], with numerous studies also examining the enhancing effects of these microbial agents on plant phosphorus uptake. However, due to the complexity of natural ecosystems, the effectiveness of these microbial agents remains uncertain [21]. Thus, how to effectively regulate the function of phosphate-solubilizing microorganisms at the plant–soil–microbe interface remains a topic for further exploration.

This paper systematically discusses the composition of phosphorus in soil and the interconversion among its components, starting from the potential of phosphorus utilization in the plant–soil system; discusses the significant role and limitations of phosphate-solubilizing microorganisms as a green biotechnological approach in enabling plants to adapt to low-phosphorus environments; proposes focusing on the interactions among soil microbes; and introduces the concept of synthetic communities to further deepen the understanding of the interactions at the plant–soil–microbe interface to mobilize insoluble phosphorus in the soil, providing theoretical guidance for the development and application of bioregulatory techniques for the efficient utilization of soil phosphorus.

## 2. Composition and Transformation of Soil Phosphorus

### 2.1. Composition of Soil Phosphorus

Soil serves as the primary reservoir of phosphorus for plant growth, with phosphorus existing in organic and inorganic forms, over 80% of which is immobile [22]. Inorganic phosphorus can be classified into mineral-bound, adsorbed, and water-soluble forms [23]. Mineral-bound phosphorus includes primary minerals such as apatite-type calcium phosphates (Ca_10_-P) and other phosphorus compounds like iron phosphates (Fe-P), aluminum phosphates (Al-P), and calcium phosphates (Ca-P) [24]. When phosphates are encapsulated by iron oxide films, they are referred to as occluded phosphorus (O-P), which represents the most abundant and biologically inaccessible form of inorganic phosphorus. Adsorbed phosphorus is associated with solid soil surfaces through van der Waals forces, chemical bonds, or phosphate anions [25] and is considered moderately active. It requires disruption by extracellular enzymes or organic acids released by biological processes to become bioavailable. Water-soluble phosphorus mainly exists as soluble phosphate ions (PO_4_^3−^, HPO_4_^2−^, and H_2_PO_4_^−^) and represents the readily available form for biological uptake [26,27]. Soil organic phosphorus generally refers to phosphorus compounds containing C-O-P or C-P bonds [28], accounting for 20–80% of total phosphorus. Its primary sources are plant and animal residues, organic fertilizers, and microbial transformation of inorganic phosphorus [29]. Organic phosphorus generally becomes available to plants through mineralization [30,31]. However, in phosphorus-deficient conditions, plants can also absorb moderately active and stable organic phosphorus [32]. Organic phosphorus can be classified into three types based on the phosphate bond: phosphoesters with C-O-P bonds, phosphonates with C-P bonds, and organic condensed phosphates (phosphoric anhydrides), which are rarely detected in soil [33]. Phosphoesters are further divided into monoesters and diesters based on the number of carbon-containing groups attached to each phosphorus atom [34]. Phosphomonoesters are chemically stable and commonly found in most soil environments, with inositol hexaphosphate (IHP) being the most prevalent [34]. Other phosphomonoesters include glycerophosphate, sugar phosphates, mononucleotides, and phosphoproteins [34]. Phosphodiesters, such as nucleic acids (RNA and DNA), phospholipids, and teichoic acids, degrade into phosphomonoesters under aerobic conditions, resulting in lower soil concentrations of phosphodiesters [35]. 2-Aminoethyl phosphonic acid (2-AEP) is the primary phosphonate in natural environments, usually found in free form or bound to lipids or macromolecules, and is easily mineralized by phosphonatase enzymes secreted by soil microorganisms [34,36,37]. Consequently, phosphonates tend to accumulate in cold, wet, or acidic environments [38]. The most important phosphoric anhydrides in nature are adenosine triphosphate (ATP) and adenosine diphosphate (ADP), which participate in energy transfer. Due to their rapid degradation to adenosine monophosphate (AMP) at ambient temperatures, they are rarely detected in natural soils [39].

### 2.2. Transformation Mechanisms of Soluble and Insoluble Phosphorus in Soil

The transformation between soluble and insoluble phosphorus in soil primarily involves two processes. The first involves the biological interconversion of organic and inorganic phosphorus. Soil organic phosphorus can be mineralized into phosphate through biochemical pathways mediated by extracellular phosphatases and microbial oxidation [40]. Some of the resulting phosphate can be immobilized by microorganisms and converted back into organic phosphorus [41]. Microorganisms play a crucial role in this process, facilitating the mineralization of organic phosphorus through nitrogen-driven enzyme metabolism and carbon-driven microbial activity [40]. Practical agricultural practices have demonstrated that microorganisms can enhance plant phosphorus utilization and accelerate phosphorus cycling [42]. When organic fertilizers are applied to farmland, specific organic phosphorus compounds are either completely converted to inorganic phosphorus, biologically immobilized, or lost from the soil [43,44].

The second process involves the adsorption, desorption, and precipitation of organic and inorganic phosphorus on the surfaces of metal oxides and clay minerals. These abiotic reactions control the mobility, transformation, and availability of phosphorus in the environment [45,46,47], influenced by factors such as mineral type and crystallinity, relative molecular mass of organic phosphorus, pH, temperature, and coexisting ions [48,49]. Mineral phosphorus dissolves through weathering, erosion, soil pH changes, ion activity, and biological actions [49,50]. The adsorption of both organic and inorganic phosphorus is affected by soil pH [25,48]. Higher pH levels increase the repulsion of phosphate anions, reducing soil adsorption capacity and increasing phosphate anion concentration in the solution. Additionally, the adsorption density of organic phosphorus on mineral surfaces decreases with increasing crystallinity and relative molecular mass of organic phosphorus [51,52]. Both water-soluble inorganic and organic phosphorus can form complexes on mineral surfaces, engage in hydrogen bonding, or precipitate on the surface [48]. However, organic phosphorus has a higher adsorption capacity and lower desorption degree than inorganic phosphorus. Iron and aluminum oxides have a stronger adsorption capacity for organic phosphorus than clay minerals [53], possibly due to the synergistic effect of organic phosphorus and metal ions promoting adsorption and fixation on mineral surfaces, especially under low pH conditions [48]. Phosphate ions are also easily adsorbed or fixed by soil metal ions (Fe^3+^, Al^3+^, Mn^2+^, and Ca^2+^); in acidic soils, they are fixed by Fe and Al oxides and hydroxides, while in alkaline soils, they form insoluble phosphorus with Ca [54]. Additionally, organic acids (e.g., humic acid, fulvic acid, and fulvic acid) can release fixed phosphorus by complexing with metal ions, inhibiting phosphorus adsorption, and the inhibition effect increases with the number of hydroxyl groups [55,56,57].

Phosphorus in soil undergoes various biogeochemical transformations. Chemical processes predominantly determine the long-term forms and distribution of soil phosphorus in most natural ecosystems [50], while biological processes primarily influence the short-term forms and distribution [58]. Consequently, biological processes are a focal point in research on phosphorus bioavailability.

Although total phosphorus (TP) content in soil is abundant, the high fixation of available phosphorus (Pi) often results in insufficient levels to meet production demands. To alleviate phosphorus limitation, chemical phosphorus fertilizers are commonly applied. However, the single-season utilization efficiency of chemical phosphorus fertilizers is low, and a significant portion of the fertilizer is easily leached into water bodies, leading to eutrophication and negatively impacting the ecological environment. Additionally, excessive fertilizer application can disrupt the balance between plant roots and microbial interactions, potentially degrading the roots’ ability to acquire phosphorus over time. Moreover, the production of chemical fertilizers is energy-intensive, and the limited reserves of phosphate rock are insufficient to support long-term fertilizer production.

## 3. Utilization Potential of Phosphorus in Plant–Soil Systems

Phosphorus (P), measured as P_2_O_5_, comprises approximately 0.2% to 1.1% of plant dry weight [59]. As an essential macronutrient that is also constrained by environmental factors, phosphorus plays a central role in photosynthesis, respiration, energy transfer, biosynthesis of macromolecules (such as ATP, nucleic acids, and proteins), and the formation of cell membranes. It is also a critical component in regulating many enzymatic reactions and signal transduction processes [60,61].

### 3.1. Mechanisms of Plant Adaptation to Low-Phosphorus Environments

Plant growth is regulated by a combination of endogenous signals and environmental factors. The root apex senses changes in soil phosphorus concentration, generating localized signals that adjust root architecture. Low-phosphorus signals in the rhizosphere suppress primary root growth and promote lateral root development [62], while internal phosphorus-deficiency signals in the plant activate phosphorus starvation responses that regulate phosphorus metabolism, transport, signaling, distribution, redistribution, and root functionality [63]. Under low-phosphorus conditions, photosynthetic products are preferentially allocated to the roots to increase the root-to-shoot ratio, thereby enhancing phosphorus uptake efficiency [64]. Additionally, some of these products are released as root exudates into the rhizosphere, such as organic acids (citric acid, malic acid, oxalic acid, etc.), amino acids and sugars (fructose, galactose, glucose, etc.) [65,66]. These exudates alter the physical and chemical properties of the soil, recruiting functional microbial communities, and enhancing the activation and utilization of soil nutrients by plants [67].

Numerous studies have demonstrated that the composition and abundance of rhizosphere microbial communities are significantly influenced by different vegetation types and soil factors. Xing et al. [68] found that even in bamboo forests with no significant differences in total soil phosphorus content, the diversity and function of phosphate-solubilizing microbial groups varied significantly, as did the levels of available phosphorus in the soil. This suggests that PSMs may play a crucial role in plant adaptation to low-phosphorus environments. Efficient phosphorus utilization is, therefore, a systematic process involving soil–plant-microbe interactions and can be improved through the selection of phosphorus-efficient plant varieties and biofertilizers. While substantial progress has been made in understanding plant mechanisms for efficient phosphorus acquisition and utilization—particularly in the areas of phosphorus metabolism [69] and the molecular, biochemical, morphological, and physiological responses of plants to phosphorus deficiency [70]—there remains a lack of understanding regarding the role of PSMs in the multi-interface interactions between plant roots, soil, and microbes. This knowledge gap limits the development of scientifically robust biofertilizers for efficient phosphorus utilization. Given that microbe-mediated phosphorus management offers both ecological and economic benefits as a sustainable approach, the synergistic mechanisms between plants and rhizosphere microbes have garnered increasing academic interest [71].

### 3.2. Synergistic Mechanisms between Rhizosphere Functional Microbiomes and Plants

The rhizosphere functional microbiome consists of a selectively recruited subset of soil microorganisms, controlled by both biotic and abiotic factors, such as plant species and soil physicochemical properties [72]. These microorganisms enhance plant growth and development by regulating nutrient uptake, mineral solubilization, disease resistance, and stress tolerance [73,74]. Plants provide a variety of substances to rhizosphere microorganisms, including amino acids, sugars, lipids, nucleic acids, growth factors, vitamins, fatty acids, organic acids, flavonoids, and enzymes (such as alkaline phosphatase, polyphenol oxidase, and α-glucosidase) [75]. These substances facilitate the colonization of soil microbial communities on plant roots, root surfaces, or within root tissues [76], thereby promoting the interaction between microorganisms and plants. Differences in root exudates among various species and genotypes promote the growth of distinct microbial branches, with certain exudates selectively attracting or repelling specific microbes, thereby shaping the rhizosphere microbiome [77]. For example, the secretion of malic acid by *Arabidopsis thaliana* roots can selectively recruit *Bacillus subtilis* depending on the amount secreted [78]. In response to nutrient stress, microorganisms exchange substances and produce signaling molecules that regulate the expression of genes in both plants and microbes, potentially driving the co-evolution of microbial communities and plants [79].

### 3.3. Types and Distribution of Phosphate-Solubilizing Functional Microorganisms

Among plant growth-promoting rhizobacteria, PSMs are key components of the phosphorus cycle and include phosphate-solubilizing bacteria, fungi, and actinomycetes [80]. Phosphate-solubilizing bacteria account for a significant portion of soil microorganisms (approximately 1–50%), whereas phosphate-solubilizing fungi constitute a smaller fraction (0.1–0.5%) [81]. Reported phosphate-solubilizing bacteria include genera such as *Bacillus*, *Pseudomonas*, *Enterobacter*, *Escherichia*, *Erwinia*, *Serratia*, and *Paenibacillus* [82], with *Bacillus* and *Pseudomonas* present in nearly all soil types [83]. Fungal genera include *Penicillium*, *Aspergillus*, and *Rhizopus* [84]. The phosphate-solubilizing ability of PSMs often diminishes or disappears during successive generations [85].

The distribution of PSMs in soil is uneven and is affected by soil physicochemical properties, vegetation type, and fertilization practices [86,87,88,89,90,91,92]. Xing et al. [68] discovered that the abundance and function of phosphate-solubilizing bacteria, such as Bacillaceae and Burkholderiaceae, vary across different bamboo forest types. Shi et al. [93] showed that the diversity of organic phosphate-solubilizing bacterial communities (oPSB), such as *Bradyrhizobium*, *Aquabacterium*, *Rhizobacter* and *Xanthomonas,* etc., is significantly influenced by soil properties, whereas the diversity of inorganic phosphate-solubilizing bacterial communities (iPSB), such as *Burkholderia*, *Azotobacter*, *Pseudomonas* and *Mycobacterium*, is not significantly affected.

### 3.4. Growth-Promoting Effects of Phosphate-Solubilizing Microorganisms in the Plant–Soil System

**Figure 2:**
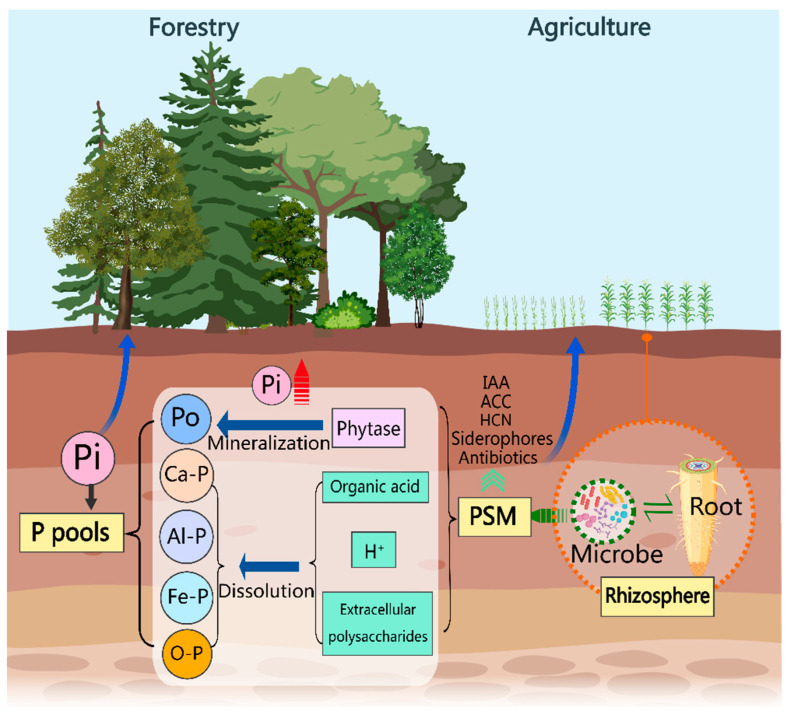
Potential for the development and utilization of phosphate-solubilizing microorganisms. Phosphate-solubilizing microorganisms (PSMs) within the rhizosphere microbiome have the potential to enhance soil phosphorus availability. These microorganisms can secrete phytases to mineralize organic phosphorus (Po) and produce organic acids, hydrogen ions, and extracellular polysaccharides to solubilize insoluble inorganic phosphorus compounds (such as Ca-P, Al-P, Fe-P, and O-P). This activity increases the concentration of available phosphorus in the soil. Additionally, PSMs can promote plant growth by secreting substances such as indole-3-acetic acid (IAA), 1-aminocyclopropane-1-carboxylate (ACC) deaminase, siderophores, and antibiotics.

Phosphate-solubilizing microorganisms convert insoluble soil phosphorus into bioavailable forms by producing phytase to mineralize organic phosphorus or secreting organic acids to dissolve inorganic phosphorus, playing a vital role in phosphorus cycling and alleviating phosphorus-deficiency-induced growth limitations in plants [18,19,94] (Figure 2). Additionally, PSMs can chelate heavy metal ions in the soil with exopolysaccharides, releasing phosphate ions directly available to plants [94]. Some PSMs also modulate plant growth by altering the concentrations of various plant hormones [95], such as indole-3-acetic acid (IAA) [96], or by synthesizing siderophores for symbiotic or non-symbiotic nitrogen fixation [97]. Moreover, PSMs can produce antibiotics, hydrogen cyanide (HCN) [98], and 1-Aminocyclopropane-1-carboxylic acid (ACC) deaminase [99], which help manage soil-borne diseases and reduce heavy metal toxicity. Therefore, PSMs with diverse plant growth-promoting and soil-improving characteristics hold significant potential as mobilizers of insoluble soil phosphorus. Research has demonstrated that the application of PSM inoculants, either alone or in combination with other plant growth-promoting microbes, can enhance phosphorus uptake, increase crop yields, and promote sustainable agriculture and environmental health. For example, Barea et al. [100] reported increased phosphorus uptake in legumes treated with phosphate rock fertilizers and inoculated with phosphorus-enhancing rhizobia, mycorrhizal fungi, and rhizobial combinations. Malviya et al. [101] found that inoculating peanut rhizosphere with a combination of phosphate-solubilizing fungi (*Aspergillus niger* and *Penicillium notatum*) significantly increased dry matter, yield, protein and oil content, and nitrogen and phosphorus levels. PSMs have also been shown to address phosphorus deficiencies in subtropical rice soils, enhancing yields [102]. Xing et al. [103] demonstrated that co-inoculating bamboo seedlings with mixed PSMs and arbuscular mycorrhizal fungi (AMF) significantly improved the conversion of insoluble soil phosphorus into available phosphorus.

Although efficient PSMs could replace chemical fertilizers and contribute significantly to reducing environmental pollution and promoting ecological balance, their effectiveness is often limited to controlled laboratory conditions and does not always translate well to complex natural environments due to local microbial and soil environmental factors [20]. Well-organized microbial communities have been found to better withstand environmental fluctuations than single strains [104]. Thus, understanding microbial interaction mechanisms is increasingly important for designing effective microbial communities.

## 4. Interactions among Soil Microorganisms

Soil is one of the most diverse microbial habitats on Earth [105]. Classical ecological theory predicts that the more overlapping the niches of two organisms, the more likely competitive behaviors will occur, leading to strong antagonistic interactions among neighboring soil microbes [106]. Soil bacteria belonging to phyla such as Acidobacteria, Verrucomicrobia, Gemmatimonadetes, and Rokubacteria have been shown to encode and produce secondary metabolites with antibacterial properties through various pathways [107]. However, cooperative traits, such as cross-feeding and degradative synergy, are also frequently detected among soil bacteria [108].

A key approach to understanding microbial interactions is to examine resource availability [109], with nutritional interactions serving as the central driving force. Under conditions of resource abundance, microorganisms compete for the same nutrients, excluding competitors, or coexist by utilizing different resources [110]. When resources are scarce, microorganisms tend to establish mutual dependencies through the exchange of metabolic products [111]. The interactions among related soil bacteria can lead to two evolutionary outcomes: resistance to antagonistic toxins and nutritional specialization to avoid antagonistic interactions [112,113]. These interactions can be categorized as neutral (0/0), positive (+/+, +/0), or negative (−/−, −/0, +/−) [114]. Therefore, from a nutritional interaction perspective, strategies can be developed to regulate the functional performance of the rhizosphere microbiome. The concept of synthetic communities (SynCom) [115], a novel research direction, has significant advantages in studying interactions among microorganisms, between microorganisms and plants, and between microorganisms and the environment.

## 5. The Role of Synthetic Communities in Plant–Soil Ecosystems

### 5.1. Introduction to the Concept of Synthetic Communities

Synthetic communities refer to microbial communities whose composition and abundance are artificially controlled and assembled. Two main design strategies are employed: the first is a “top-down” approach, where environmental perturbations (such as changes in environmental factors or the addition of compounds) are used to manipulate microbial communities to achieve desired functions [116]. The second is a “bottom-up” approach, which involves reconstructing microbial communities by highlighting metabolic network interactions at the molecular level through the use of metabolic network design software and reaction models to evaluate their stability [112]. However, both methods currently face different technical challenges and there are still many gaps to be filled [117].

### 5.2. Strategies for Constructing Synthetic Communities

Constructing synthetic communities requires a foundation of high-throughput sequencing data, making it essential to explore the interactions within the rhizosphere microbiome or between it and other microbial communities [118]. However, the specific roles and mechanisms of these interactions in community assembly remain unclear [119,120]. The rhizosphere microbiome is a highly complex and dynamic community, and factors such as the sequence of species colonization during microbial assembly, along with environmental changes in light, temperature, and humidity, can significantly alter community composition [121,122,123,124,125,126]. Xun et al. [72] categorized the rhizosphere microbiome into environment-driven and plant-genetic-driven groups based on different assembly mechanisms of the rhizosphere microbiome. This classification facilitates researchers in filtering redundant information from high-throughput sequences according to their needs, thereby highlighting key groups within microbial communities. The refined approach allows for a more targeted analysis that emphasizes the pivotal taxa within the microbial consortia.

The core microbiome, a key component of the rhizosphere microbiome, is a stable group more likely to influence host physiology and phenotype in natural environments [127]. Simplifying the rhizosphere microbiome to its core components to investigate their key functions under controlled conditions may provide a fundamental approach to understanding how microbial interactions affect their functions [128]. However, due to technological limitations and cultivation methods, current research primarily focuses on high-abundance species. Co-cultivation on high-throughput or traditional media can increase the production and diversity of microbial secondary metabolites to some extent, thereby exploring microbial interactions [121]. This approach is commonly used to construct synthetic communities and examine their regulatory roles in rhizosphere microbial functions.

### 5.3. Applications of Synthetic Communities

Several studies have utilized synthetic communities to investigate microbial interactions within ecosystems. For instance, Castrillo et al. [111] explored the molecular mechanisms by which Arabidopsis prioritizes nutrient stress responses over defense mechanisms using synthetic communities isolated from its rhizosphere. Sun et al. [129] demonstrated through metabolic modeling, omics analysis, and functional gene knockout experiments that applying Bacillus SQR9, in conjunction with native *Pseudomonas stutzeri*, forms a stable mixed-species biofilm in the rhizosphere, promoting plant growth through metabolic cross-feeding. Liu et al. [130] found that synthetic communities composed of aluminum-tolerant strains from the rice rhizosphere can mitigate soil acidification and aluminum toxicity in acidic fields, improve the utilization of residual phosphorus in the topsoil, and enhance rice yields. Other studies have developed synthetic communities to improve tea quality [131], degrade soil organic pollutants or heavy metals [132,133], resist the invasion of plant roots by soil pathogens [134], and enhance crop stress resistance [135]. These findings indicate that synthetic communities hold significant potential for regulating microbial functions in plant–soil systems, and the rational design of highly effective phosphate-solubilizing synthetic communities could play a key role in enhancing phosphorus mobilization functions in the rhizosphere microbiome.

## 6. Conclusion and Future Perspectives

In conclusion, designing synthetic communities with efficient phosphate-solubilizing functions by exploring interactions among phosphate-solubilizing microorganisms presents a viable strategy for mobilizing insoluble soil phosphorus, alleviating phosphorus limitations in agricultural and forestry production, and promoting plant growth and yield. However, further research is needed to refine the methods for constructing synthetic communities, deepen the understanding of microbial interactions, and elucidate the roles of phosphate-solubilizing microorganisms in the multi-interface interactions within the plant root-soil–microbe system. Understanding how synthetic communities can positively impact plant–soil systems will provide better guidance for developing biological control technologies to optimize phosphorus utilization.

Future research should focus on several key areas, including (1) the stability of synthetic communities, which is related to the interactions among community members. At a small scale, stability is based on the coexistence of community members and their cooperative interactions. At a larger scale, antagonistic interactions among microorganisms can also enhance stability by promoting diversity [136]. Further exploration of microbial interactions at different interfaces will be crucial for enhancing the effectiveness of synthetic communities. (2) The effects of co-inoculation of synthetic communities with other organic matter on community functionality. The “top-down” design of synthetic communities manipulates microbial functions through environmental disturbances; therefore, co-inoculating synthetic communities with disturbance factors may enhance their effectiveness. (3) New methods for constructing synthetic microbial consortia. Since both “top-down” and “bottom-up” approaches have limitations, a recently proposed method by Ruan et al. [137] combines both strategies to modify natural microbial communities and design functionally enhanced synthetic consortia. Additionally, emerging tools such as gene editing for precise microbial engineering provide a foundation for designing stable and efficient multi-species synthetic communities in the future.
